# Stent-Graft Fabrics Incorporating a Specific Corona Ready to Fenestrate

**DOI:** 10.3390/ma16144913

**Published:** 2023-07-09

**Authors:** Jing Lin, Xiaoning Guan, Mark Nutley, Jean M. Panneton, Ze Zhang, Robert Guidoin, Lu Wang

**Affiliations:** 1 Key Laboratory of Textile Science and Technology, Ministry of Education, College of Textiles, Donghua University, Shanghai 201620, China; jlin@dhu.edu.cn (J.L.); guanxiaoning@dhu.edu.cn (X.G.); 2 Key Laboratory of Textile Industry for Biomedical Textile Materials and Technology, Donghua University, Shanghai 201620, China; 3 Division of Vascular Surgery and Department of Diagnostic Imaging, University of Calgary, Peter Lougheed Centre, Calgary, AB T2N1N4, Canada; 4 Division of Vascular Surgery, Eastern Virginia Medical School, Norfolk, VA 23501, USA; 5 Department of Surgery, Faculty of Medicine, Université Laval and Centre de Recherche du CHU de Québec, Québec, QC G1V 0A6, Canada

**Keywords:** in situ fenestration, stent-graft, fabrics, balloon angioplasty, tearing

## Abstract

In situ fenestration of endovascular stent-grafts has become a mainstream bailout technique to treat complex emergent aneurysms while maintaining native anatomical visceral and aortic arch blood supplies. Fabric tearing from creating the in situ fenestration using balloon angioplasty may extend beyond the intended diameter over time. Further tearing may result from the physiologic pulsatile motion at the branching site. A resultant endoleak at the fenestrated sites in stent-grafts could ultimately lead to re-pressurization of the aortic sac and, eventually, rupture. In an attempt to address this challenge, plain woven fabrics were designed. They hold a specific corona surrounding a square-shaped cluster with a plain weave fabric structure, a 2/2 twill, or a honeycomb. The corona was designed to stop potential further tearing of the fabric caused by the initial balloon angioplasty and stent or later post-implantation motion. The cluster within the corona was designed with relatively loose fabric structures (plain weave, 2/2 twill weave, and honeycomb) to facilitate the laser fenestration. Two commercial devices, Anaconda (Vascutek, Terumo Aortic) and Zenith TX2 (Cook), were selected as controls for comparison against this new design. All the specimens were characterized by morphology, thickness, and water permeability. The results demonstrated that all specimens with a low thickness and water permeability satisfied the requirements for a stent graft material that would be low profile and resistant to endoleaks. The in situ fenestrations were performed on all fabrics utilizing an Excimer laser followed by balloon angioplasty. The fabrics were further observed by light microscopy and scanning electron microscopy. The dimension of the fenestrated apertures was smaller than the balloon’s diameter. The tearing was effectively confined within the corona. The clinical acceptability of this concept deserves additional bench testing and animal experimentation.

## 1. Introduction

**Figure 1:**
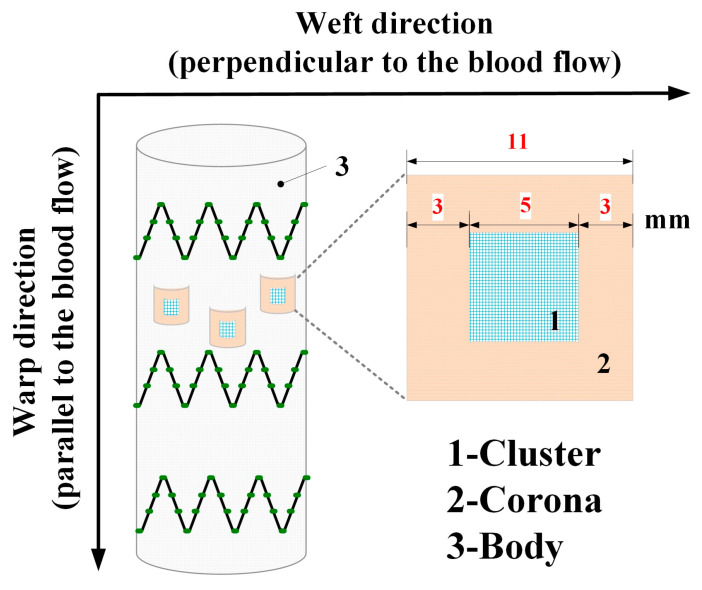
Sketch of an in situ fenestrated stent-graft with a tubular fabric with the specific “corona”, which consisted of the body and the corona. A square “cluster” (5 mm in width) was incorporated into the square corona (11 mm in width) and surrounded by it. The function of the corona is to prevent the tearing caused by the balloon’s dilation, while the cluster allows for easy balloon dilation by the surgeon.

In-situ fenestration of stent grafts is now accepted worldwide as a bailout option for frail or elderly patients presenting with emergent, life-threatening complex aortopathies who are only candidates for minimally invasive endovascular aneurysm repair (EVAR) [1,2]. The success of this technique compares favorably to the chimney and other similar methods [3,4]. Although considerable refinements of the technique of in-situ laser fenestration have been achieved, the mainstream manufacturers of stent-graft devices have not yet overcome the challenge of developing and producing optimized and commercially available fabrics designed to be fenestrated in situ [5]. The in-situ fenestration procedure is performed by first penetrating the fabrics of the stent-grafts by laser fenestration, followed by balloon angioplasty. This is considered the optimal technique by most stent-graft manufacturers [6]. Alternative fenestration techniques (with and without laser) have recently been investigated through the AARCHIF registry in the cardiac literature [7]. All the fenestration techniques for in situ endovascular aortic arch repair are viable treatment options [8]. Regrettably, adverse clinical outcomes were observed due to embolic stroke or type III endoleaks [9,10]. The various types of woven fabrics currently selected by most commercial stent-graft manufacturers include 4/4 twill woven monofilament, plain woven multifilament, and fancy warp-locked (plain + warp double) multifilament yarns. These materials permit fenestration of the fabrics by either energy fenestration (laser or radiofrequency) or mechanical fenestration (needles) to create the initial orifice necessary to enable balloon insertion and dilation [11,12,13]. Using current knowledge, surgeons select the most appropriate technique based on their skills, the imaging equipment available, and the availability of devices that allow safe and reliable fenestrations [14]. In response to balloon dilation before the placement of covered stents, the risk of fabric tearing with the current stent-graft fabric designs still needs to be optimized to eliminate the risk of endoleak and aortic sac size progression [15]. The success of the fenestration of fabrics is highest when using a non-compliant balloon whose diameter is 8 mm or less. The use of cutting balloons in this context is no longer recommended [6]. The issue of tear propagation post-ballooning of the fabric and after branch graft flow restoration has been addressed previously [16,17]. The goal of this research was to develop and optimize a unique fabric structure to prevent the tearing of the stent graft (Figure 1) caused by the necessity to first perform balloon angioplasty during the in situ fenestration of the stent graft while performing urgent endovascular treatment of complex aortic pathology.

## 2. Materials and Methods

### 2.1. Yarn Selection

Medical-grade polyester multifilament fibers (RxFiber LLC, Windsor, CA, USA) were selected to weave the fabrics. The specifications of the yarn 40D/27f were as follows: 27 fibers in each yarn and a 40 deniers yarn’s liner density. (The denier is the unit of fineness for yarn equal to the fineness of a yarn weighing one gram for every 9000 m). The diameter of the fiber is 12.33 ± 0.32 μm. Such a specification is similar to that of the fiber employed in the Anaconda stent-graft material (Vascutek, Terumo Aortic, Inchinnan, Scotland, UK) at 12.87 ± 0.64 μm).

### 2.2. Design and Manufacture of Flat Fabrics Incorporating a Corona

**Figure 2:**
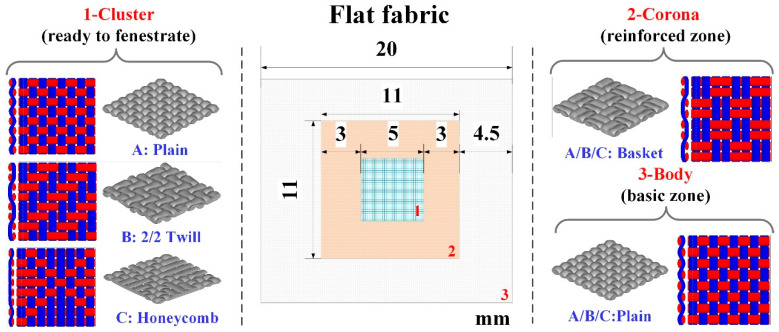
The configuration and structure of the flat fabric. The plain weave is selected for the body (basic zone) as common device, while the basket weave is selected for the corona (reinforced zone) in order to enhance the structure and prevent tearing. There are three kinds of weaves (plain, 2/2 twill, and honeycomb) selected as the cluster (fenestrated zone) to be penetrated easily by the laser probe or needles. However, isolate the blood flow from the aneurysm effectively.

The basic fabric, designed to integrate a cluster within a corona, was a plain woven body 20 cm in width and a selected choice in length. The fabric count was 7 ends/mm and 7 picks/mm in all the prototypes. The coronas were created to stop tearing after fenestration and balloon angioplasty using non-compliant balloons. This fabric structure was a basket, 11 mm × 11 mm. The clusters were declined in three different constructions: plain weave (A), 2/2 twill weave (B), and honeycomb (C) in squares 5 mm × 5 mm within the coronas. The initial plain fabric without a corona and a cluster served as a reference (D). All the specimens were woven on a customized shuttle loom at Donghua University. The thermal treatment was performed after the weaving with a temperature setting of 180 °C for 10 min (Figure 2, Table 1).

Two commercial stent-grafts, Anaconda (Vascutek, Terumo Aortic, Inchinnan, Scotland, UK) and Zenith TX2 (Cook Medical, Bloomington, IN, USA), were selected as reference endografts.

### 2.3. Fabric Characteristics

#### 2.3.1. Thickness

It was measured according to ISO Standard 7198 [18] to confirm that the thicknesses of the fabrics with the corona were similar to those of the control D and to assess that potential future commercial devices could be inserted into delivery sheaths of diameters equivalent to or even smaller than those of currently available commercial devices. Each zone of the specimen was measured three times, and then the mean and standard deviation were calculated.

#### 2.3.2. Water Permeability

**Figure 3:**
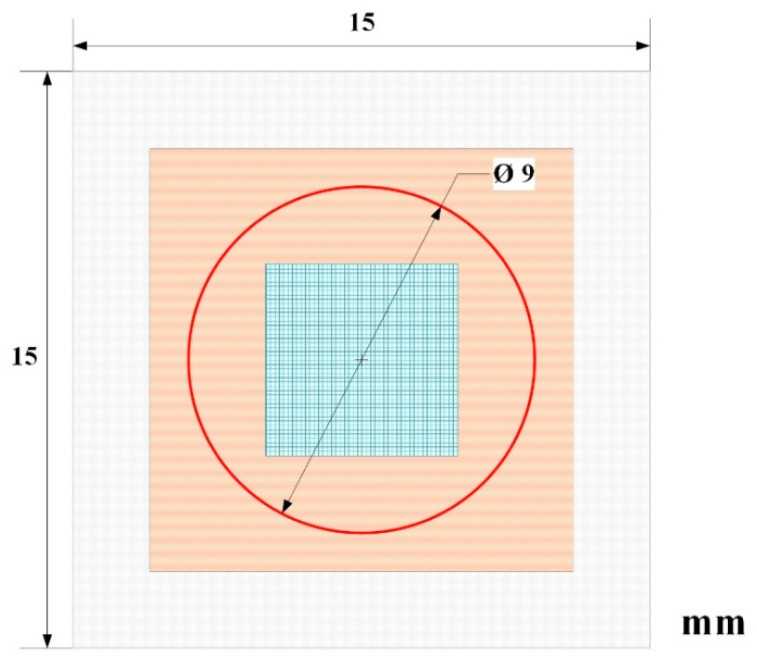
Schematic illustrating configuration of the specimen for locally water permeability test. The water flow will penetrate the surface areas (red cycle) of the fabric.

The water flow volume is measured through the flat fabric wall per centimeter square at 16 kPa, i.e., 120 mmHg pressure per minute (mL/min/cm^2^) [19]. Each fabric specimen was cut into a square of 15 mm × 15 mm^2^. The coronas fitted with the clusters were included for specimens A, B, and C. The total effective surface area (*A*) of fabrics was 0.636 cm^2^ (Ø 9 mm) (Figure 3). The volume of distilled water (*Q_i_*) per minute was collected continuously over ten minutes to calculate each fabric’s average water permeability (*P*) according to the following Equation (1). The water permeability test was performed three times for each specimen.

(1) $$P_{i}=\frac{Q_{i}}{A}\;\left(i=1,\;2,\;\ldots \ldots ,10\right)$$

### 2.4. Fenestrations

#### 2.4.1. In-Situ Fenestration

Four series of fabric perforations were made on the experimental devices A, B, and C, the control fabric D, and the commercial device fabrics using the 308-nm CVX-300 Excimer Laser System (Spectranetics, Colorado Springs, CO, USA), which can successfully and efficiently create apertures in polyester-based stent-grafts outside the manufacturer’s instructions for use (IFU). The laser operates at a wavelength of 308 nm. A Turbo Elite Laser Ablation Catheter (2.3 mm diameter) at high energy (i.e., fluency of 60 mJ/mm^2^) was used to create each fenestration [4,9]. The fabrics were submerged in the bottom of a basin filled with a physiologic saline solution at room temperature. The fenestrations were performed in three lines of five punctures for each cluster design: the first one was selected as the control; the other two were dilated by non-compliant balloons (Mustang Over-The-Wire PTA Balloon Dilatation Catheters, Boston Scientific, Marlborough, MA, USA) of 6 mm and 8 mm diameter, respectively. They were inflated to a nominal pressure of 10 atmospheres. The same protocol was followed for the control and commercial devices.

#### 2.4.2. Observations in Microscopy

The fabrics were examined non-destructively as received, after laser fenestration, and after ballooning. Each one was observed at 20× magnification with a light compound microscope (SMZ745T, Nikon Imaging (China) Sales Co. Ltd., Shanghai, China) fitted with a CCD camera (Digital Sight DS-Fil, Nikon Imaging (China) Sales Co. Ltd., Shanghai, China). Then square areas of 15 mm × 15 mm^2^ holding the clusters were cut out of the fabric, gently flattened, and fixed on a stub without tension using conductive adhesive. They were then sputter-coated with platinum and observed at 100× magnification by the scanning electron microscope (Quanta 250 FEI Company, Hillsboro, OR, USA) at a 15 kV accelerating voltage. The images were processed with Adobe Photoshop CS.

#### 2.4.3. Dimension of the Fenestrated Apertures

The area and the maximal tearing length of each fenestrated aperture were measured under optical microscopy and scanning electron microscopy. Adobe Photoshop CS measured the lengths of the fenestrations in the warp (parallel to the direction of the blood flow) and weft (perpendicular to the direction of the blood flow) directions. They were defined as the maximal length between the two extremities of the holes in both warp and weft directions. Ultimately, the area of fenestration was measured. The data were analyzed by SPSS version 26 for Windows (SPSS Inc., Chicago, IL, USA). All results were expressed as the mean ± standard deviation (SD). The significance between the data groups was tested using the One-way ANOVA test. A value of *p* < 0.05 was considered significant.

## 3. Results

### 3.1. Stent-Graft Fabrics for In Situ Fenestration

**Figure 4:**
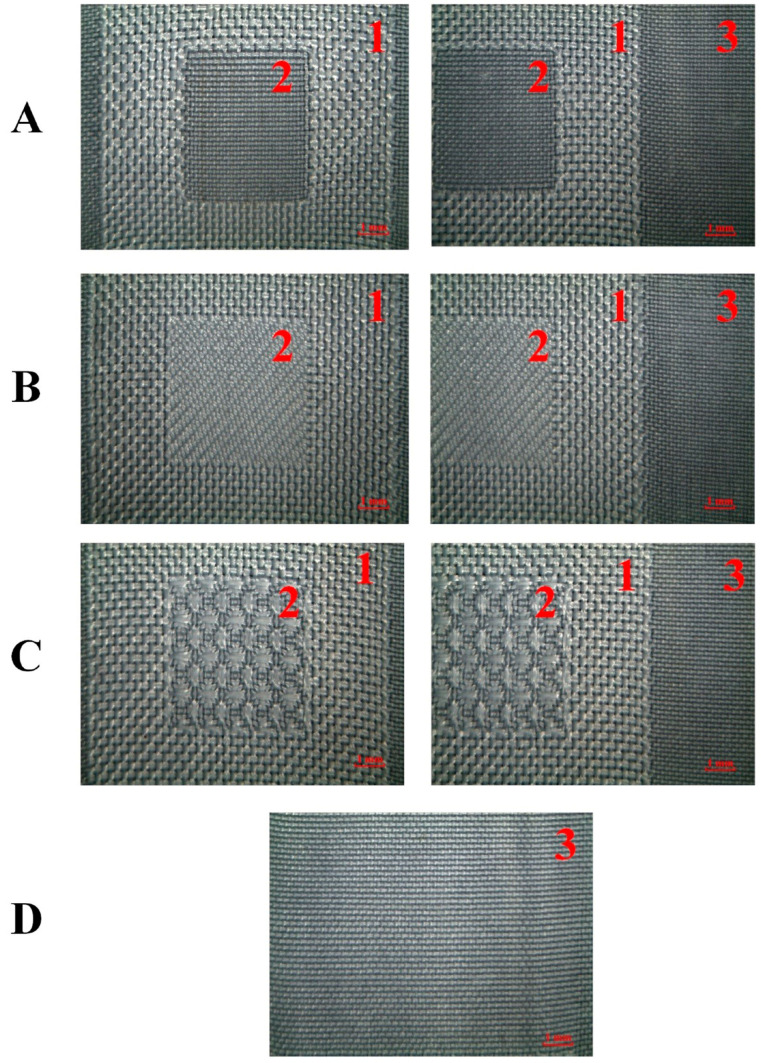
Gross observation of three flat fabrics (**A**–**C**) incorporating a specific corona (2, reinforced zone), the cluster (1, fenestrated zone), and the body (2, basic zone). The fabric (**D**) was the control with the only basic zone (3).

The fabrics incorporated different zones, i.e., body, corona, and cluster. They were flat. The structure of the basic zone and reinforcement zones’ structures was uniform and stable. The boundary transition between different zones was well-defined. The control specimen D with the plain weave showed a structure much denser than the other two structures, the corona and the cluster. The structure of 2/2 twill with the right diagonals was observed on the cluster of specimen B, while the honeycomb shapes were present on specimen C with the honeycomb weave. There were no skewed weft yarns observed on all specimens. Therefore, the three-stage gradient fabric with stable structures was obtained by the integrated textile preparation technology with varying structures (Figure 4).

### 3.2. Fabric Characteristics

#### 3.2.1. Thickness

**Figure 5:**
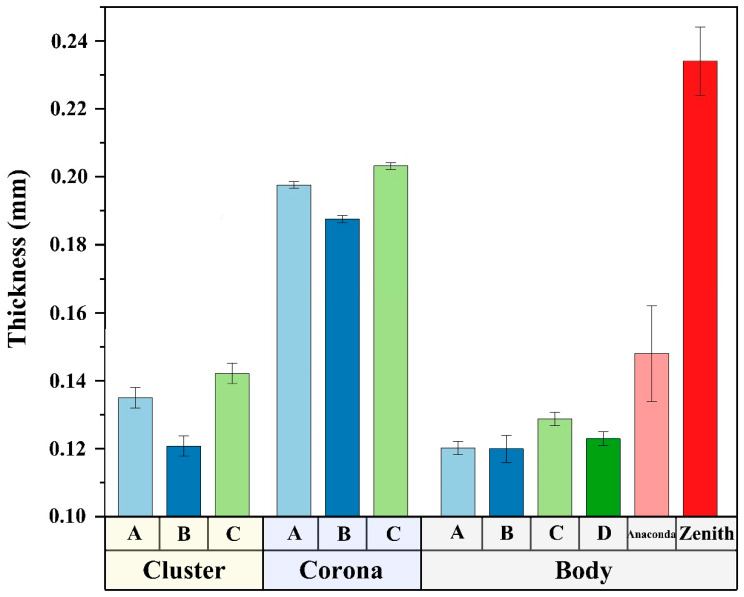
Thickness of the fabrics. The thickness of the Zenith is almost more than twice of all specimens in body (basic zone). The corona (reinforced zone) is thicker than the body and the cluster (fenestrated zone) due to the prevention of further tearing of the fabric in the fenestrated zone. Specimens A, B, and C have different constructions in the clusters—plain weave, 2/2 twill weave, and honeycomb, respectively. Specimen D is a plain fabric serving as a reference.

All the specimens with the reinforced zone (corona) and zones to be fenestrated A, B, and C were thicker than the control D (0.123 ± 0.002 mm). Meanwhile, the thickness of the corona zone was higher than that of the basic zone and zones to be fenestrated. Fabric specimen C (reinforced zone: 0.203 ± 0.002 mm) was the thickest, while B (0.188 ± 0.002 mm) was the thinnest. The Zenith reference TX2 fabric was the thickest at 0.234 ± 0.010 mm (Figure 5).

#### 3.2.2. Water Permeability

**Figure 6:**
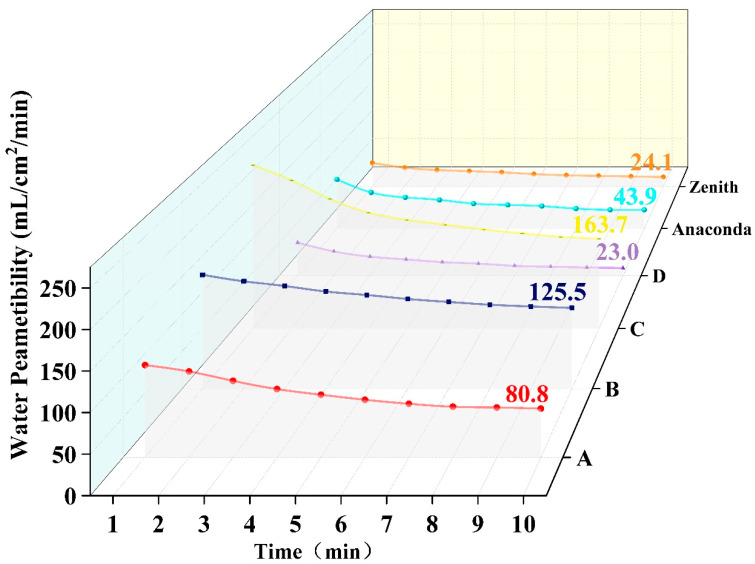
Locally, water permeability. Specimens A and B are preferred to be selected as their water permeability is lower than specimen C’s. A decrease in water permeability with time is observed.

It decreased with time from 0 to 10 min (Figure 6). Within the reinforced and fenestrated zones, the water permeability of specimens was higher than control D (23.0 ± 2.6 mL/min/cm^2^). Specimen C (163.7 ± 17.2 mL/min/cm^2^) was the highest, while specimen A (80.8 ± 2.6 mL/min/cm^2^) showed the lowest permeability. The water permeability of the commercial references Anaconda and Zenith TX2 measured 43.9 ± 4.6 mL/min/cm^2^ and 24.1 ± 2.4 mL/min/cm^2^, respectively.

### 3.3. In-Situ Fenestration

#### 3.3.1. Gross Observations

**Figure 7:**
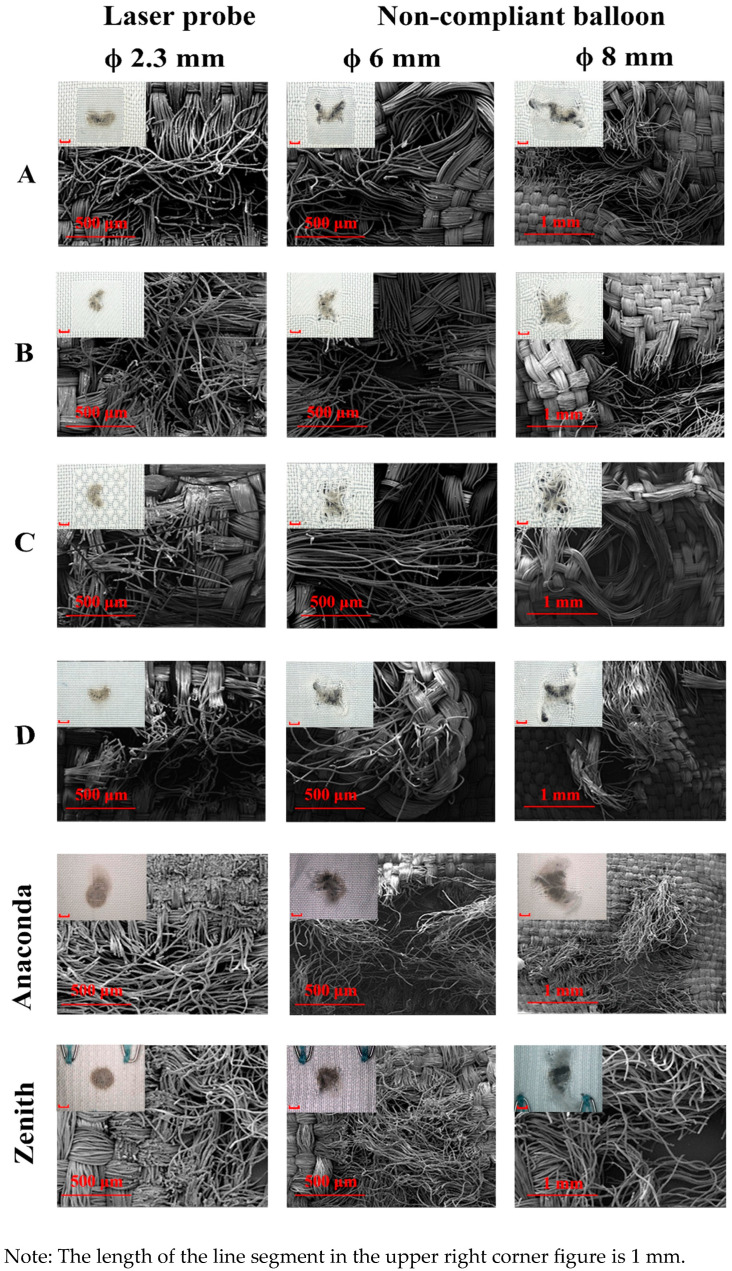
Light microscopy and SEM photo of laser fenestrations after the non-compliant balloon deployment of specimens and commercial references. It is obviously observed that the corona (reinforced zone) could prevent the further tearing of the dilation of the non-complaint balloon in the cluster (fenestrated zone).

The dilations with non-compliant balloons permitted enlargement of the initial laser perforation (Figure 7). Compared to control D, the corona effectively prevented further tearing. Among the specimens, C presented larger apertures than A and B. Specimen C, with the honeycomb weave in the fenestrated zones, demonstrated a longer float length of yarns while the interweave points of yarns were less, which resulted in the least amount of uncontrolled tearing. Thus, it demonstrated the highest risk of causing excessive tearing.

The yarns within the cluster were broken by the balloon dilatation instead of cutting away from the fabric. When the balloon was removed, the broken but flexible yarns could partially recoil back to their original position. Thus, the shape of the fenestrated apertures of specimens B and C looked to a “butterfly”. Acutely, the shape of the apertures of specimen B most closely approximated a concentric circle with a similar tearing length in both directions. Furthermore, it demonstrated that the yarns broken within the cluster (fenestrated zone) were destroyed by the balloon dilations. However, it is observed that the yarns within the corona (reinforced zone) only curled along the balloon dilation direction instead of breaking. This suggests that, due to the reinforced fabric structure, the yarns in the corona could prevent further tearing.

#### 3.3.2. The Area and the Length of Fenestrated Apertures

**Figure 8:**
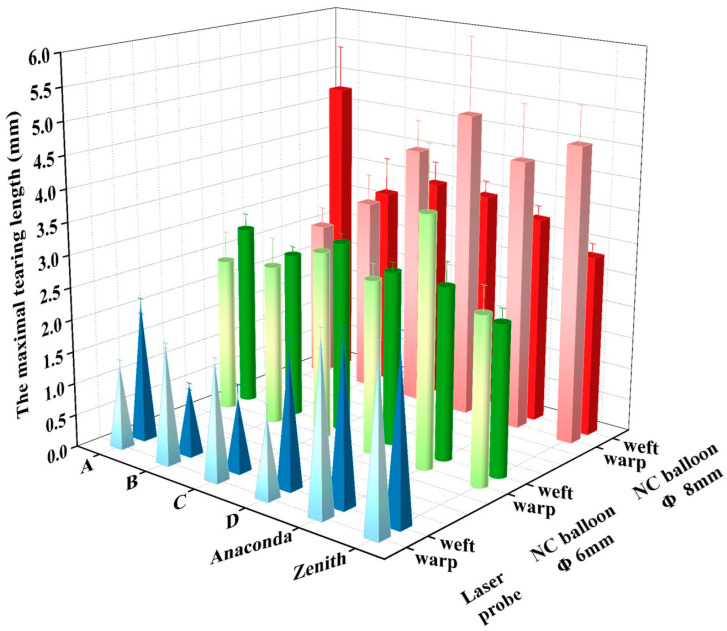
The maximal tearing length in warp and weft directions of specimens and commercial references. It is presented that the maximal tearing length of the fenestrated apertures of the specimens with the specific corona is shorter than the specimen D, Anaconda, and Zenith.

Table 2 and Figure 8 show the maximal length and area of the fenestrated apertures. Compared to specimen D, the samples with the reinforced zones have lower tearing lengths and areas. Specimen C was the largest among the three specimens, while B had the shortest tearing length and smallest area post-balloon dilatation (*p* < 0.05). Furthermore, the tearing directions in the warp and weft of Specimen B were similar when using the non-compliant balloons (*p* < 0.05).

Compared to the Anaconda, the tearing length and the area of the apertures of specimen B are smaller (*p* < 0.05). The Zenith has a double warp structure; thus, its tearing length (2.83 ± 0.18) in the weft direction is shorter than all specimens (*p* < 0.05). However, its tearing length (4.62 ± 0.58 mm) in the warp direction is longer than that of specimen B (3.08 ± 0.45 mm) (*p* < 0.01).

## 4. Discussion

The issue of post-fenestration fabric tearing has been raised in the past [20,21,22,23] and has been more recently addressed in several patents that were released [24,25] (Table 3). The primary purpose of this research was to create a fabric designed to make the initial perforation easier and decrease the risk of fabric tearing caused by the balloon angioplasty. The selection of an optimal fabric weave or tightening the structure of the weaves are two options to solve the tearing issue within the fenestration regions of the fabrics. These results demonstrate a potential solution to this problem. However, much work must be done to translate this new fabric design into a fully functioning aortic endograft for in vivo animal or human testing.

The construction of a specific cluster nested within a corona of a woven textile fabric has now been successfully achieved. This novel fabric design may effectively eliminate the risk of progressive dilation over time and blood leakage past the fenestrations’ site. The water permeability results of this fabric are similar to the anticoagulated whole blood permeability. The water permeability of the novel specimens was higher than that of the commercial devices; however, it was still much lower than 300 mL/min/cm^2,^ which could prevent type IV endoleaks [19].

Furthermore, the clinical acceptability of this concept deserves additional bench testing and animal experimentation. To be clinically adopted and implemented, a stent-graft incorporating such a sophisticated fabric must demonstrate its benefits for patients and physicians. At a minimum, it must not be inferior to the current technology and must demonstrate clinical durability over the long term [26,27,28]. The degree of success of any new implantable device can be expressed by the 3Bs that we have previously highlighted: bifunctionality, biodurability, and biocompatibility. We will address these in order of importance as they apply to this new technological development.

1. Biofunctionality. It is anticipated that the wall of the device will be thin enough to fit into a delivery sheath whose diameter is as narrow as possible [29]. The thickness of the body of the fabrics created for this study, A, B, C, and D, at about 0.12 mm, was in the same range as the Anaconda fabric and less than half of the one of Zenith TX2. The corona thickness was maintained between the Anaconda and the Zenith TX2. The clusters were thinner. Thus, the thickness of the flat fabrics satisfied the clinical requirement of being low profile, which could be compressed into the delivery system of a small diameter delivery system to be used even in patients with smaller iliac arteries. The next step will be to incorporate seamless sleeves in between stents. The devices will then need to include radiopaque markers to facilitate tracking and positioning of the fenestration sites to locate and puncture the center of the clusters under fluoroscopy. Optimization of the location of these clusters on the main body of the stent-graft has already been done during the development of the Cook CT branch stent-graft. They observed about 100 typical CTAs of patients with aortic pathology and determined the location of the visceral branches with 83 to 86% success [30,31]. A future “In-situ Specific Endograft” would be designed in a similar manner. Still, instead of branches, clusters would be placed at the most likely positions of the visceral arteries, for example. The stent-graft would likely be designed with the SMA fenestration already created so that when the stent-graft is initially partially deployed into the aorta, all visceral vessels will continue to be perfused. The last vessel to be cannulated through this single pre-manufactured fenestration would be the open SMA artery, completing the seal of blood flow from the aortic sac while minimizing visceral ischemic time during the procedure. Another feature of this design may be the incorporation of a pre-canulation wire built into the SMA fenestration within the delivery device. This would allow the insertion of a stabilization wire into the SMA at the beginning of the deployment of the device to allow both for orientation of the endograft and also to ensure that the SMA can be easily stented near the end of the procedure as the last visceral vessel to be treated for the above-stated reasons. The development of more precise imaging techniques will also facilitate this intervention [32]. Ultimately, manufacturing devices available for off-the-shelf usage will become feasible as the range of diameters required to serve side branches is limited. However, there is still a long way to go.

In situ fenestration remains a bailout technique, and intensive research to achieve better optimization of the procedure with the development of more sophisticated fabric structures of the main body of the endograft remains paramount. To create the initial orifices, clinicians can select between energy fenestration (laser or radiofrequency) and mechanical fenestration (needles) [33,34,35]. Lasers are restricted to polyester fabrics, whereas directional needles can be used in all polymeric sleeves, including expanded polytetrafluoroethylene. The production of hazardous chemicals created by lasers in the fenestration of ePTFE remains a serious concern [36]. The following fenestrations can be safely achieved with non-compliant balloons 6 or 8 mm in diameter, as the tearing cannot extend beyond the limits of the corona [37,38,39,40,41,42]. Balloons of larger diameters are likely to cause significant and irregular tears, leading to an increased risk of endoleaks.

2. Biodurability. This characteristic allows the device to maintain biofunctionality in the long term, i.e., maintaining blood supply to the downstream end organs. There must be a minimal risk of endoleak development. The branching orifice will remain a site of weakness unless an appropriate design for a branch graft interface becomes commercially available. As the site of fenestration is seriously constrained in the corona, extensive tearing is highly unlikely based on the specific structure of the corona.

The polyester sleeve becomes impervious shortly after deployment, and blood oozing is prevented based on the capacity of the polyester to hold blood’s flow and the blood’s properties [43,44]. This is the result of the wicking characteristics of the polyester yarns. For the reasons mentioned above, the selection of multifilament yarns is considerably advantageous compared to monofilaments. In the absence of iatrogenic damages caused by suturing the stents or during the insertion of the device in the sheaths, the imperviousness of the polyester sleeve is undisputable, even in the absence of fabric encapsulation. The branch must maintain its position at the site of fenestration to ensure blood flow without kinking [45]. Practically, the diameter of the branch shall be moderately wider in diameter than the orifice of the vessel being treated, and the size selected will extrude approximately 10 to 15% into the aortic lumen. In summary, any risk of endoleakage or kinking within the branch grafts, risking thrombosis, must be eliminated.

3. Blood compatibility. Very little attention is paid to the biocompatibility of stent grafts. Encapsulation of the walls of the stent-grafts remains an enigma [46]. Good biointegration would mean the development of an external capsule penetrating the interstices between the yarns. Such a capsule would be made of collagen and elastin sinusoidal fibers. At best, an external capsule consists of stretched fibers of collagen and elastin that transform the blood conduit into a pipe devoid of compliance [47]. The development of an internal capsule whose flow surface is endothelialized represents an ideal that remains out of reach [48]. At best, some endothelium can develop a few millimeters in continuity with the aorta endothelium. The capture and integration of endothelial cells from the flowing blood are insufficient [49]. Practically, there is no connection between the internal and external capsules. As observed in animals, transmural communication at a subcellular level cannot play a critical role in fall-out-based endothelialization [50]. New strategies for improving blood compatibility and endothelialization of cardiovascular devices are being developed. The challenge of achieving this goal is immense, although research in this area has continued for decades [51].

As priority is given to maintaining graft integrity and guaranteeing blood flow, discovering a perfect blood-compatible surface soon is very unlikely based on the complexities involved [52].

## 5. Conclusions

The successful design of novel fabrics incorporating the corona (reinforced zone) as well as a cluster (fenestrated zone) resulted in a thickness equivalent to or even less than the Zenith graft fabric but with much higher resistance to tearing. Prototypes A and B with fenestrated and reinforced zones effectively prevented fabric tearing caused by dilation with the non-compliant balloons. With fabric thickness being a primary consideration, the reinforced prototype B with the plain weave for the body (basic zone), the basket weave for the corona, and the 2/2 twill weave for the cluster may be the optimal choice. However, such an innovative fabric with a safe, specific “corona,” specially designed into a tubular sleeve fitted with stents, deserves additional validation in bench studies and animal experimentation.

## Figures and Tables

**Table 1:** The information of the novel design of fabrics.

Design	1. Cluster	2. Corona	3. Body
Zones	Site of fenestration	Reinforced zone	Basic zone
Function	Facilitates the perforation by the laser probes or needles	Improves the tearing resistance caused by the dilation of balloons	Guarantees its imperviousness to prevent blood oozing
Fabric structure	● Specimen A: Plain weave	Specimens A, B and C: Basket	Specimens A, B, and C: Plain
	● Specimen B: 2/2 twill		
	● Specimen C: Honeycomb		

**Table 2:** The maximal tearing length and area of the fenestrated apertures.

No.	Fenestration Devices		Maximal Tearing Length (mm)		Area (mm^2^)
			Warp	Weft	
A	Laser probe		1.26 ± 0.11	2.10 ± 0.12	1.94 ± 0.34
	NC balloon (mm)	Ø 6	2.42 ± 0.45	2.84 ± 0.23	4.32 ± 0.51
		Ø 8	2.52 ± 0.29	4.76 ± 0.69	7.00 ± 0.63
B	Laser probe		1.82 ± 0.04	1.06 ± 0.05	1.74 ± 0.09
	NC balloon (mm)	Ø 6	2.52 ± 0.43	2.60 ± 0.12	2.98 ± 0.58
		Ø 8	3.08 ± 0.45	3.16 ± 0.56	5.54 ± 1.47
C	Laser probe		1.78 ± 0.08	1.1 ± 0.23	1.96 ± 0.42
	NC balloon (mm)	Ø 6	2.94 ± 0.09	2.98 ± 0.13	4.54 ± 0.93
		Ø 8	4.10 ± 0.46	3.48 ± 0.33	7.98 ± 1.67
D	Laser probe		1.20 ± 0.20	2.04 ± 0.25	1.92 ± 0.11
	NC balloon (mm)	Ø 6	2.70 ± 0.23	2.72 ± 0.13	4.96 ± 0.44
		Ø 8	4.80 ± 1.19	3.44 ± 0.22	9.12 ± 1.33
Anaconda	Laser probe		2.60 ± 0.15	2.49 ± 0.14	5.14 ± 0.87
	NC balloon (mm)	Ø 6	3.88 ± 0.99	2.69 ± 0.36	6.71 ± 1.80
		Ø 8	4.23 ± 0.86	3.24 ± 0.19	12.13 ± 3.85
Zenith	Laser probe		2.60 ± 0.20	2.52 ± 0.29	5.05 ± 0.61
	NC balloon (mm)	Ø 6	2.60 ± 0.40	2.35 ± 0.20	6.27 ± 0.82
		Ø 8	4.62 ± 0.58	2.83 ± 0.18	12.77 ± 3.25

Note: NC stands for the non-compliant.

**Table 3:** The comparison among the patents referred to regarding in-situ fenestration grafts.

Patent	Materials	Method	Object	Potential Clinical Issues
CN107119371B 27 September 2019 Donghua University	Textile strands	Two various fabric weaves (basket weave for corona and the 2/2 twill weave for cluster) at fenestrated regions.	To make the perforation easier. To prevent the tearing after the balloon dilation. No permeability of the designed fenestrated regions if the fenestration is not operated.	Absence of radiopaque marker to facilitate tracking the fenestrated regions.
US 8597342B2 3 December 2013 Cook Medical Technologies LLC	Textile strands	Reduce the yarn density ^#^ at designed fenestrated regions.	To make the perforation easier.	Tearing of fabric after balloon dilation. Risks of endoleak in the vicinity of fenestration orifices or the higher porosity caused by reduced yarn density. Delayed type IV endoleak.
US 8353943B2 15 January 2013 Cook Medical Technologies LLC	Textile strands plus metal strands	Reduce the yarn density at designed fenestrated regions. Form a tighter structure around a branched vessel stent.	To make the perforation easier. To reinforce the fenestration orifice and reduce the possible fluid leakage.	The stiff metal stands might stop balloon dilation and increase the graft rigidity. Abrasion of the metal stands in one direction to the textile strands in another direction.

^#^ Yarn density means the number of yarns in per 10 cm in warp or weft direction of woven fabric.

## Data Availability

Data are contained within this article.
